# The role of food processing in the inflammatory potential of diet during pregnancy

**DOI:** 10.11606/S1518-8787.2019053001154

**Published:** 2019-12-16

**Authors:** Carolina Assis Silva, Izabela da Silva Santos, Nitin Shivappa, James R. Hebert, Lívia Castro Crivellenti, Daniela Saes Sartorelli

**Affiliations:** I Universidade de São Paulo, Faculdade de Medicina de Ribeirão Preto, Curso de Nutrição e Metabolismo. Ribeirão Preto, SP, Brasil; II Universidade de São Paulo, Faculdade de Medicina de Ribeirão Preto, Programa de Pós-Graduação em Nutrição e Metabolismo. Ribeirão Preto, SP, Brasil; III University of South Carolina, Arnold School of Public Health, Department of Epidemiology and Biostatistics. Columbia, SC, USA; IV Universidade de São Paulo, Faculdade de Medicina de Ribeirão Preto, Programa de Pós-Graduação em Saúde Pública. Ribeirão Preto, SP, Brasil; V Universidade de São Paulo, Faculdade de Medicina de Ribeirão Preto, Departamento de Medicina Social. Ribeirão Preto, SP, Brasil

**Keywords:** Prenatal Nutrition, Food Consumption, Energy Intake, Industrialized Foods

## Abstract

The aim was to investigate the relationship between the energy contribution (E%) of foods according to the degree of industrial processing and the energy-adjusted dietary inflammatory index (E-DII) in pregnancy. Two 24-hour dietary recalls were obtained from each of the 784 pregnant women. Adjusted linear regression models allowed observing an inverse association between E-DII scores and E% from minimally processed foods β = -0.049 (95%CI -0.055– -0.042) and a direct association with the E% of ultra-processed foods β = 0.052 (95%CI 0.045–0.058), indicating a relationship between the dietary inflammatory potential and the degree of industrial processing of foods.

## INTRODUCTION

Diet is a strong determinant of chronic inflammation. To quantify the role of diet in modulating inflammation, a known cause of numerous chronic diseases, Shivappa et al. developed the dietary inflammatory index (DII^®^)^1^, based on the results of 1,943 scientific studies that investigated the effect of dietary intake on six inflammatory markers. Evidence suggests a pro-inflammatory maternal diet in pregnancy is associated with a higher risk of low birth weight and greater neonatal adiposity^2 , 3^.

The increase in the prevalence of chronic diseases in the last few decades can be partially attributed to dietary patterns based on the substitution of the consumption of meals prepared with unprocessed or minimally processed foods for the consumption of ultra-processed products^4^. A high energy percentage (E%) of the diet from ultra-processed foods is related to greater energy density, saturated and *trans* fat content, and added sugar, lower fiber and micronutrient content^4^, all of which are attributes of pro-inflammatory diets^1^. Conversely, diets with higher E% from unprocessed or minimally processed foods are rich in micronutrients with anti-inflammatory potential^1 , 4^, suggesting a possible association between the degree of industrial processing of foods and the inflammatory potential of the diet. However, no study has investigated this relationship.

This study aimed to investigate the relationship between the E% of unprocessed or minimally processed foods, processed foods, and ultra-processed foods and the DII of the diet of pregnant women.

## METHODS

This was a secondary analysis of data from a cross-sectional study conducted with 784 adult pregnant women receiving prenatal care in Primary Health Units (PHUs) in the city of Ribeirão Preto, São Paulo, between 2011 and 2012. Women were interviewed between 24 and 39 weeks of gestation, as described in detail by Barbieiri et al^5^. This study was approved by the Research Ethics Committee of the School Health Center of the Ribeirão Preto Medical School (075/2015-CEP/CSE-FMRP-USP) of the University of São Paulo. Pregnant women agreeing to participate in the study signed the informed consent form.

The diet was evaluated using two 24-hour dietary recalls (24h-R) obtained on nonconsecutive days, using the “multiple-pass” technique, with a mean of ten days between replications. The second 24h-R had a response rate of 73%, which is considered acceptable to investigate the relationship between diet and health outcomes.

The NutWin^a^ program was used to estimate the nutritional value of the foods. The Brazilian Food Composition Table ( *Tabela Brasileira de Composição Química dos Alimentos* – TACO)^b^, the Brazilian Table of Food Composition ( *Tabela Brasileira de Composição de Alimentos* – TBCA)^c^, and the Table of the United States Department of Agriculture Research Service (USDA)^d^ were used to estimate the intake of nutrients.

Foods were classified according to the NOVA criteria^4^. In this study, the E% of the diet from the consumption of unprocessed or minimally processed foods, processed foods, and ultra-processed foods was estimated. Handmade preparations were classified as they were consumed considering the classification of their main ingredient, i.e., the classification for risotto is the same as that for rice.

The Multiple Source Method (MSM)^e^ was used to estimate the usual intake of energy and nutrients and the consumption of foods. The Goldberg method was adopted for the estimation of under-reporting of energy intake (EI), considering the ratio EI:BMR ≤ 1.35.

To estimate the dietary inflammatory index (DII^®^), 36 of its possible 45 components were used; garlic, onion, ginger, clove, turmeric, saffron, pepper, oregano, and rosemary were not accurately obtained in the 24h-R. The algorithm to estimate the DII involves several steps. The individuals’ dietary intake is first standardized to a global database and then converted to centered proportions^1^. Each food parameter-specific proportion is then multiplied by the respective literature-derived inflammatory effect score to get the food parameter-specific DII score, which are then summed up to obtain the overall DII score for an individual^1^. Furthermore, we estimated energy-adjusted DII (E-DII) scores by converting food parameters from dietary intake to amount per 1000 kcal. To compute the E-DII scores, we relied on an energy-adjusted global database.

Sociodemographic information was obtained using a structured questionnaire, and the Brazilian Economic Classification Criterion was used for the economic classification. For the pre-pregnancy BMI calculation, the pregestational weight recorded in the pregnant woman’s medical records was considered, and the height was measured by trained interviewers.

The relationship between the E% of the diet from foods according to the degree of industrial processing and the E-DII was investigated using linear regression models. Models fit with E-DII (continuous) as the dependent variable were adjusted for age, schooling, socioeconomic class, pre-pregnancy BMI, and energy under-reporting. The residuals of the models were normally distributed. *P* < 0.05 was considered significant, and the R Core Team (2016)^f^ was used for the analyses.

## RESULTS

The mean age (SD) of the pregnant women was 28 (5) years, and the mean (SD) energy intake was 2053 (518) kcal. Under-reporting of energy intake was observed in 47% of the study participants. The mean (SD) of the E% from unprocessed or minimally processed foods, processed foods, and ultra-processed foods was 48 (13), 13 (7), and 32 (13), respectively. The mean (SD) of the overall DII score was 2.24 (1.34), ranging from -3.35 to 5.44. After adjusting for energy intake (i.e., E-DII), it was 1.70 (1.36), ranging between -2.66 and +5.96.

In the adjusted linear regression models, an inverse association was observed between the E% of the unprocessed or minimally processed foods of the diet and the E-DII. Conversely, a direct association was observed between the E% of the ultra-processed foods of the diet and the E-DII ( Table 1 ). No association was found between the %E of processed foods and E-DII.

**Table 1:** Association between the consumption of unprocessed or minimally processed foods, processed foods, and ultra-processed foods and the energy-adjusted Dietary Inflammatory Index (E-DII) score in pregnant women. Ribeirão Preto, SP, Brazil. *n* =784.

Food groups	β	95%CI		R^2^
*Unprocessed or minimally processed foods (E%)*				
Unadjusted model*	-0.058	-0.065	-0.053	0.33
Adjusted model †	-0.049	-0.055	-0.042	0.31
*Processed foods (E%)*				
Unadjusted model*	0.001	-0.013	0.014	0.01
Adjusted model †	0.006	-0.007	0.019	0.11
*Ultra-processed foods (E%)*				
Unadjusted model*	0.056	0.050	0.062	0.28
Adjusted model †	0.052	0.045	.0.058	0.32

* Linear regression models adopting the energy-adjusted dietary inflammatory index (E-DII) as the dependent variable.

† Models adjusted for age (years), education (years of schooling), social class (from E to A), pre-pregnancy BMI (kg/m^2^), and energy under-reporting (yes/no).

## DISCUSSION

In this study, a direct association between the E% of ultra-processed food products and a greater pro-inflammatory potential of pregnant women’s diet was observed. Conversely, a higher E% of unprocessed or minimally processed foods was directly associated with a greater anti-inflammatory potential of the diet.

Ultra-processed foods are nutrient sparse and have high energy density^4^, which are both attributes of a pro-inflammatory diet^1^. However, minimally processed foods are rich in micronutrients^4^ with a high anti-inflammatory potential^1^.

Evidence suggests increased consumption of ultra-processed products is a relevant risk factor for the development of chronic noncommunicable diseases, such as obesity, metabolic syndrome, and associated diseases^4^. Chronic, low-grade systemic inflammation is a common determinant of these pathologies^1^. The findings of this study indicate that the association between the consumption of ultra-processed food products and the occurrence of chronic diseases may be mediated by subclinical inflammatory processes.

Previous studies have suggested a maternal diet with greater pro-inflammatory potential is associated with a higher risk of low birth weight and greater neonatal adiposity^2 , 3^. A plausible explanation for the fetal growth deficit could be maternal vascular impairment because of inflammation, resulting in reduced blood flow to the fetus^2^. Alternatively, a pro-inflammatory diet may trigger oxidative stress, increasing the lipid oxidation and the release of free fatty acids into the plasma, which in turn may lead to fetal lipotoxicity and increased neonatal adiposity^3^.

The DII/E-DII scores of pregnant women’s diet varies widely among studies. In American pregnant women, a variation of the mean of the DII score was observed between -2.6 and +1.4^2^ and between +0.4 and +1.5^3^. In the two studies, DII scores were estimated from 27 and 28 food parameters, respectively^2 , 3^. In this study, the mean DII ranged from -3.35 to +5.44, with greater variation between the maximum anti-inflammatory and the maximum pro-inflammatory scores. These discrepancies may be inherent in differences in the sociocultural context of food choices, especially at this stage of the life cycle. Another reason could be that DII scores were estimated from a larger number of food parameters (36) in the current study compared with the other two (i.e., 27 and 28).

The cross-sectional design, the aspects inherent in studies that use food consumption data, and the absence of biochemical markers for inflammation are among the limitations of this study. Since data were previously obtained to the NOVA classification system, some misclassifications might have occurred. Similar to previous studies conducted in pregnant women, a high proportion of energy under-reporting was found. Moreover, data on nine of the 45 components of the DII were not available. However, among the strengths of this study, it was unprecedented in investigating the inflammatory potential of the diet according to the degree of industrial processing of foods consumed during pregnancy. Data from this study indicate a direct association between the E% from ultra-processed products and a greater pro-inflammatory potential of the diet, as well as a direct association between the E% of unprocessed or minimally processed foods and a higher anti-inflammatory potential of pregnant women’s diet. Future tests of these hypotheses should be undertaken using various study designs.

## Footnotes

a NutWin – Nutritional Support Program. Version 1.5. São Paulo: Paulista School of Medicine; 2002.

b Universidade Estadual de Campinas, Núcleo de Estudos e Pesquisas em Alimentação. Tabela brasileira de composição de alimentos – TACO. 2. ed. Campinas, SP: NEPA; 2006 [cited 2018 Nov 1]. Available from: http://www.crn1.org.br/wp-content/uploads/2015/04/taco.pdf?x53725

c Universidade de São Paulo, Faculdade de Ciências Farmacêuticas, Departamento de Alimentos e Nutrição Experimental.Tabela brasileira de composição de alimentos – TBCA. Version 6.0. São Paulo; 2017 [cited 2018 Nov 1]. Available from: http://www.fcf.usp.br/tbca/

d USDA National Nutrient Database for Standard Reference. Beltsville: U.S. Department of Agriculture, Agricultural Research Service; 2001 [cited 2018 Nov 1]. Available from: https://www.ars.usda.gov/northeast-area/beltsville-md/beltsville-human-nutrition-research-center/nutrient-data-laboratory/docs/sr14-home-page

e German Institute of Human Nutrition Postdam-Rehbrücke, Department of Epidemiology. The Multiple Source Method (MSM). Potsdam Rehbrücke (DEU): DIFE; c2008-2011 [cited 2018 Nov 1]. Available from: https://msm.dife.de/

f R Core Team. R: a language and environment for statistical computing. Vienna: R Foundation for Statistical Computing; 2015 [cited 2018 Nov 1]. Available from: https://www.R-project.org
